# Dehydroepiandrosterone supplementation and the impact of follicular fluid metabolome and cytokinome profiles in poor ovarian responders

**DOI:** 10.1186/s13048-023-01166-6

**Published:** 2023-06-02

**Authors:** Veronique Viardot-Foucault, Jieliang Zhou, Dexi Bi, Yoshihiko Takinami, Jerry. K. Y. Chan, Yie Hou Lee

**Affiliations:** 1 Department of Reproductive Medicine, KK Women’s and Children’s Hospital, 100 Bukit Timah Road, Singapore, 229899 Singapore; 2 Translational ‘Omics and Biomarkers Group, KK Research Centre, KK Women’s and Children’s Hospital, 100 Bukit Timah Road, Singapore, 229899 Singapore; 3 Department of Pathology, Shanghai Tenth People’s Hospital, Tongji University School of Medicine, Shanghai, 200072 China; 4 Bruker Japan, 3-9 Yokohama City, Kanagawa, 220-0022 Japan; 5 Present Address: Kanomax Analytical Incorportated, Shimizu Suita City, Osaka Japan; 6 Obstetrics and Gynaecology Academic Clinical Program, Duke-NUS Medical School, 8 College Road, Singapore, 169857 Singapore; 7 Singapore-MIT Alliance for Research and Technoology, 1 CREATE Way, Singapore, 138602 Singapore

**Keywords:** Metabolomics, Follicular fluid, DHEA, Poor ovarian responder, Cytokines

## Abstract

**Background:**

Poor ovarian responders (POR) are women undergoing in-vitro fertilization who respond poorly to ovarian stimulation, resulting in the retrieval of lower number of oocytes, and subsequently lower pregnancy rates. The follicular fluid (FF) provides a crucial microenvironment for the proper development of follicles and oocytes through tightly controlled metabolism and cell signaling. Androgens such as dehydroepiandrosterone (DHEA) have been proposed to alter the POR follicular microenvironment, but the impact DHEA imposes on the FF metabolome and cytokine profiles is unknown. Therefore, the objective of this study is to profile and identify metabolomic changes in the FF with DHEA supplementation in POR patients.

**Methods:**

FF samples collected from 52 POR patients who underwent IVF with DHEA supplementation (DHEA +) and without (DHEA-; controls) were analyzed using untargeted liquid chromatography-tandem mass spectrometry (LC–MS/MS) metabolomics and a large-scale multiplex suspension immunoassay covering 65 cytokines, chemokines and growth factors. Multivariate statistical modelling by partial least squares-discriminant regression (PLSR) analysis was performed for revealing metabolome-scale differences. Further, differential metabolite analysis between the two groups was performed by PLSR β-coefficient regression analysis and Student’s t-test.

**Results:**

Untargeted metabolomics identified 118 FF metabolites of diverse chemistries and concentrations which spanned three orders of magnitude. They include metabolic products highly associated with ovarian function – amino acids for regulating pH and osmolarity, lipids such fatty acids and cholesterols for oocyte maturation, and glucocorticoids for ovarian steroidogenesis. Four metabolites, namely, glycerophosphocholine, linoleic acid, progesterone, and valine were significantly lower in DHEA + relative to DHEA- (*p* < 0.05–0.005). The area under the curves of progesterone glycerophosphocholine, linoleic acid and valine are 0.711, 0.730, 0.785 and 0.818 (*p* < 0.05–0.01). In DHEA + patients, progesterone positively correlated with IGF-1 (Pearson r: 0.6757, *p* < 0.01); glycerophosphocholine negatively correlated with AMH (Pearson r: -0.5815; *p* < 0.05); linoleic acid correlated with estradiol and IGF-1 (Pearson r: 0.7016 and 0.8203, respectively; *p* < 0.01 for both).

In DHEA- patients, valine negatively correlated with serum-free testosterone (Pearson r: -0.8774; *p* < 0.0001). Using the large-scale immunoassay of 45 cytokines, we observed significantly lower MCP1, IFNγ, LIF and VEGF-D levels in DHEA + relative to DHEA.

**Conclusions:**

In POR patients, DHEA supplementation altered the FF metabolome and cytokine profile. The identified four FF metabolites that significantly changed with DHEA may provide information for titrating and monitoring individual DHEA supplementation.

**Supplementary Information:**

The online version contains supplementary material available at 10.1186/s13048-023-01166-6.

## Background

Poor ovarian responders (POR) are a sub-group of infertile women that account for 9–26% of in vitro fertilization (IVF) indications [1, 2]. In patients designated as “poor responders,” so-called due to poor response to ovarian stimulation given during IVF workup, the limited number of obtained oocytes remains the major problem in optimizing the live birth rates [3]. As a result of a lower number of oocytes retrieved, there are fewer embryos to select and transfer, and subsequently these patients have lower pregnancy rates per transfer and lower cumulative pregnancy rates per started cycle compared with normal responders. In PORs the mechanism of ovarian insufficiency can be multifactorial with causes such as ovarian surgery especially in case of endometrioma [4, 5], uterine artery embolization for the treatment of uterine leiomyoma [6, 7], genetic defects, chemotherapy, radiotherapy, autoimmune disorders, single ovary, chronic smoking [8, 9], or linked to diseases such as diabetes mellitus Type I [10]. However, in most cases, follicular depletion plausibly reflecting premature ovarian aging [11], clinically translates into a reduction of implantation rates, an increase of early pregnancy loss, and disappointingly low IVF success [12, 13].

In each menstrual cycle, human ovaries produce a single dominant follicle. Growth of the dominant follicle encompasses enlargement of the oocyte, replication of follicular cells, and formation and expansion of a fluid-filled follicular antrum or cavity, providing a specialized microenvironment for the development of oocytes. Follicular fluid (FF) that fills the antrum cavity is derived from the surrounding theca capillaries, abundant and easily accessible during IVF procedures due to ample volume being produced during follicle maturation [14]. FF are rich in metabolites, notably hormones, amino acids and lipids that are critical for oocyte growth and development, which determines subsequent potential to achieve fertilization and embryo development [15]. As such, constituents of the FF surrounding the oocyte provides a unique biochemical window to the growth and differentiation of the oocyte [16]. To unravel the biochemical composition of human FF and its impact on oocyte development, metabolomic analyses using gas chromatography–mass spectrometric and proton nuclear magnetic resonance metabolomic analyses have been conducted [17–22], as were proteomic analyses [23–28]. These studies mainly report the FF profiles of IVF patients, whereas the FF metabolome of POR remains poorly characterized. Furthermore, the effect of dehydroepiandrosterone (DHEA) on the FF metabolome has not been previously studied.

DHEA is a steroid produced in the adrenal cortex and the ovarian theca cells in women that is converted into more active forms of androgens such as testosterone [5]. It has been suggested that DHEA supplementation may increase the number of available follicles in PORs through an increased serum level of insulin-like growth factor, increased follicular response to follicle stimulating hormone (FSH), shifts to aerobic metabolism [29] and improved quality of oocytes [30]. However, the efficacy of DHEA pre-treatment has been controversial, with partial to reasonable clinical evidence being observed [31–37]. Based on these findings, we conducted a study to evaluate whether the FF metabolome differed in POR patients treated with DHEA or not, and whether the FF metabolome may be predictive of IVF outcome. Furthermore, DHEA has immunoregulatory functions [38], and a large-scale study of FF cytokines was conducted in parallel to reveal DHEA immunomodulatory targets.

## Material and methods

### Ethical approval and study population

The local Institutional Review Board approved the study (CIRB/2011/404/D) and written informed consent was obtained from each participant. A prospective case–control study was conducted to evaluate the metabolic and cytokine effect of DHEA administration in women, below the age of 42 starting their IVF treatment who met one of the two following features of POR (an abnormal ovarian reserve test and/or a previous poor response to ovarian stimulation in an IVF cycle) were assessed for eligibility [3]. The ESHRE working group on Poor Ovarian Response Definition of diminished ovarian reserves (AMH < 1.0 ng/mL or Day 2 or 3 FSH > 10 IU/L), or women with fewer than four oocytes retrieved with either standard long or antagonist protocols was used to defined POR in this study [3]. Inclusion criteria included women with diminished ovarian reserves (anti-müllerian hormone < 1.0 ng/mL or D2/3 follicle stimulating hormone > 10 IU/L), or women with fewer than four oocytes retrieved with either standard long or antagonist protocols. The study excluded women with previous or current DHEA supplementation, use of corticosteroids within the past three months, major systemic illnesses, and allergy to DHEA, women with women BMI > 37.5. A total of 60 subjects was enrolled into the study. 30 eligible patients received DHEA (Pharma Natural, USA) at the dose of 75 mg/day for three to eight months prior starting their controlled ovarian stimulation (COS), herein known as DHEA + , and 30 patients who received no treatment enrolled into the study (DHEA-; controls), but two DHEA + and six control patients did not complete the study. In DHEA + , two patients were withdrawn for stopping their DHEA treatment 4 and 5 months before their IVF treatment. In DHEA-, six subjects were withdrawn as they have postponed or stop further infertility treatment. Therefore, the study was conducted for 28 patients in the DHEA + group and 24 in the control DHEA- group. Table 1 summarizes the baseline characteristics of the patients in this study. Average age (DHEA-: 36 years; DHEA + , 37 years) and body mass index (DHEA-: 24 kg/m^2^; DHEA + , 23 kg/m^2^) were similar in both groups (*p* > 0.05). Baseline hormones between the two groups were comparable.

**Table 1:** Baseline characteristics of poor ovarian responder patients in this study

	**DHEA+ group** **(*n* = 28)**	**DHEA- group (*n* = 24)**	***P*-value†**
Age (year), mean (SD)	37 (3)	36 (4)	0.3087
BMI (kg/m^2^), mean (SD)	23 (5)	24 (5)	0.4755
Race, *n* (%)			0.0014
Chinese	21 (75.0)	14 (58.3)	
Malay	0 (0.0)	8 (33.3)	
Indian	1 (3.6)	2 (8.4)	
Others	6 (21.4)	0 (0.0)	
Primary infertility, *n* (%)			0.3117
Yes	19 (67.9)	13 (54.2)	
No	9 (32.1)	11 (45.8)	
Infertility duration (years), median (range)	4 (1-16)	4 (1-11)	>0.9999
Primary infertility diagnosis, *n* (%)			0.7688
Male factor	22 (78.6)	17 (70.8)	
Tubal factor	1 (3.6)	1 (4.2)	
Endometriosis	1 (3.6)	2 (8.3)	
Low ovarian reserve	3 (10.6)	4 (16.7)	
Others	1 (3.6)	0 (0.0)	
Cycle number* n* (%)			0.0088
Cycle 1	4 (14.3)	12 (50.0)	
Cycle 2 & above	22 (78.6)	12 (50.0)	
Basal FSH (IU/L) , mean (range)^a^	8.4 (4.8-27.7)	6.5 (3.6-16.6)	0.1176
Estradiol (pmol/L) , mean (range)^a^	98.7 (37.0-320.0)	92.4 (37.0-273.0)	0.5432
AMH (ng/ml) , mean (range)^a^	0.7 (0.2-2.8)	0.8 (0.2-2.7)	0.0783
Free Testosterone (pmol/L), mean (range)^a^	2.0 (0.5-14.3)	1.7 (0.9-2.9)	0.1364
DHEA-S (µmol/L), mean (range)^a^	4.0 (0.5-17.3)	3.8 (0.9-10.0)	0.3178
Antral follicle count, mean (range)^a^	4.6 (1-15)	5.1 (0-12)	0.018
Ovarian volume RO (cm^3^), mean (range)^a^	7.3 (1.9-24.0)	7.7 (2.0-29.5)	0.4636
Ovarian volume LO (cm^3^), mean (range)^a^	5.1 (1.8-16.6)	8.2 (2.4-26.7)	<0.0001

^a^Blood baseline parameters, antral follicle count and ovarian volumes were obtained in 27 patients in the DHEA group and 20 patients in the control group and were summarized by geometric mean (range)

†Student's t-test was used for continous data and chi-square used for categorical data, which are race, primary infertility, infertility years, primary infertility diagnosis and cycle number

### IVF/ICSI protocol

All individuals received the same stimulation protocol, same starting dose of gonadotropin, and fertilization technique. Briefly, the IVF/ICSI treatment cycle involved an antagonist-based COS protocol consisting of daily sub-cutaneous injections of recombinant-FSH (Puregon, Follitropin beta, 300iu; MSD, USA) and highly-purified human menopausal gonadotropin (Menopur; Menotropin, 150 IU; Ferring Pharmaceuticals, Germany) with initiation of gonadotropin releasing hormone antagonist (Ganirelix, Orgalutan, 0.25 mg s/c; MSD, USA) on day 5 of COS. The dose of Menopur and Puregon could be further increased depending on individual ovarian response. All patients had this standardized antagonist (short) protocol: no agonist (long) protocol was used. Human chorionic gonadotropin (i.m 10,000 IU hCG; Pregnyl; MSD, USA) was administered when at least one follicle measured ≥ 17 mm in diameter (averaged orthogonal measurements). The endometrial thickness, peak estradiol and progesterone levels were assessed on the day of human chorionic gonadotropin (hCG) trigger. Ultrasound-guided trans-vaginal oocyte retrieval was performed 36 h after hCG administration. The effect of DHEA supplementation on the markers of ovarian reserve (anti-müllerian hormone; AMH), follicular function (IGF-1), ovarian follicular levels of estradiol, testosterone, and DHEA, collected from the lead follicle at the time of OPU were assessed through ELISA as previously described [39].

Embryo transfer was performed on day 2 or day 3 of embryo-culture, and luteal phase support was achieved with vaginal progesterone (micronized progesterone, Utrogestan, 200 mg three times a day, Besins-International, France). Pregnancy was established by serum beta-hCG seventeen days post embryo transfer. Clinical pregnancy will be established by a transvaginal ultrasound four weeks after embryo transfer. IVF/ICSI clinical and hormonal outcomes are shown in Table 2. There was no significant difference in clinical pregnancy, number of oocytes retrieved, metaphase II oocytes or number of embryos transferred.

**Table 2:** Primary and secondary outcomes between DHEA+ and DHEA - control groups

	**DHEA + group**	**DHEA- control group**	**Effect estimate**^**a**^	***p*-value**
	**(*n* = 28)**	**(*n* = 24)**	**(95% CI)**	
**Clinical outcomes**				
Clinical pregnancy, *n* (%)	2 (7.1)	3 (12.5)	0.57 (0.10–3.14)	0.652
No. of oocytes retrieved, mean (SD)	5.2 (2.9)	4.5 (3.6)	0.7 (-1.4 to 2.9)	0.507
No. of metaphase II oocytes, mean (SD)	4.0 (2.5)	3.5 (2.2)	0.6 (-1.0 to 2.1)	0.459
No. of embryos, mean (SD)	2.7 (2.4)	2.3 (1.8)	0.4 (-1.1 to 1.8)	0.601
Embryos transferred,* n* (%)				
0	5 (23.8)	3 (17.7)	1	
1	9 (42.9)	5 (29.4)	1.03 (0.53 to 2.00)	1
2	7 (33.3)	9 (52.9)	0.78 (0.44 to 1.39)	0.667
**Hormonal outcomes**^**b**^				
DHEA-S (µg/ml)	870.73 (96.00–3385.57)	182.45 (47.25–589.19)	4.77 (2.83 to 8.04)	< 0.001
Free Testosterone (pg/ml)	87.68 (19.54–457.86)	25.50 (11.56–202.89)	3.44 (2.12 to 5.59)	< 0.001
Estradiol (× 10^5^) (pg/ml)	17.2 (6.15–137.00)	14.7 (2.15–32.5)	1.17 (0.67 to 2.02)	0.571
AMH (ng/ml)	1.37 (0.21–12.60)	1.56 (0.39–6.12)	0.88 (0.45 to 1.70)	0.687
IGF1 (ng/ml)	0.23 (0.01–18.84)	0.23 (0.08–14.22)	0.99 (0.37 to 2.63)	0.976

^a^The effect estimate refers to relative risk for clinical pregnancy, absolute mean difference for cycle outcomes and relative mean difference for hormonal biomarkers (ratio of geometric means)

^b^Hormones’ concentration in follicular fluid of the leading follicle, are summarized by geometric mean (range)

### Sample preparation

FF (DHEA + , *N* = 18 and DHEA-, *N* = 16) were divided for metabolomics and cytokine analyses. For untargeted metabolomics analysis, sample preparation followed previously published reports with some modifications [40, 41]. A volume of 50 µL from each FF sample was thawed at 4 °C, and FF proteins were precipitated with 200 µL ice-cold methanol. After vortexing, the mixture was centrifuged at 16,000 rpm for 10 min at 4 °C and the supernatant was collected and evaporated to dryness in a speedvacuum evaporator. The dry extracts were then re-dissolved in 200 µL of water/methanol (98:2; v/v) for liquid chromatography-tandem mass spectrometry (LC–MS/MS) analysis.

A pooled quality control (QC) sample was generated to allow comparison of analytic behavior over long periods of time. The pooled reference samples were for the purposes of quality control (i.e., to ensure relative consistency among identical samples within days) and for quality assurance (i.e., to ensure consistent results between days). They did not contribute data to downstream statistical analysis.

### Liquid Chromatography-Tandem Mass Spectrometry-based Metabolomics

The supernatant fraction from sample preparation step was analyzed using Agilent 1290 ultra-high pressure (performance) liquid chromatography system (Waldbronn, Germany) equipped with a Bruker impact II Q-TOF mass spectrometer with its normal electrospray ionization (ESI) ion source (Bruker Daltonics). 2.5 μL of samples was injected and were separated using Waters Acquity HSS T3 (2.1 mm i.d. × 100 mm, 1.8 µm) at a flow rate of 0.2 mL/min. The oven temperature was set at 50 °C. The gradient elution involved a mobile phase consisting of (A) 0.1% formic acid in water and (B) 0.1% formic acid in methanol. The initial condition was set at 5% B. A 5.5 min linear gradient to 60% B was applied, followed by a 13 min gradient to 98% B (total 24 min including wash and re-equilibration) at a flow rate of 0.4 ml/min. The ion spray voltage was set at 4,500 V, and the Dry Temperature was maintained at 150 °C. The drying nitrogen gas flow rate and the nebulizer gas pressure were set at 8.0 L/min and 26 psi, respectively. Calibration of the system was performed using sodium formate clusters before data acquisition. The stability of the LC–MS method was examined and evaluated by sodium formate clusters (1 mM NaOH, 0.1% formic acid, 50% 2-propanol) infused into the system.

The ESI mass spectra were acquired in positive ion mode. Mass data were collected between *m/z* 100 and 1000 at a rate of three scans per second. Auto MS/MS was triggered at 8 Hz with duty cycle of 1.5 s. Threshold was set at 1500 counts, with active exclusion activated after 3 spectra, released after 0.3 min and overwritten if the current or previous intensity changes. MS/MS spectra were acquired at collision energy of 20–50 eV automatically varied by the charge states and the intensities of the selected precursors. Fragment spectra acquisition was carried out at a scan rate dependent on the MS precursor intensities—MS/MS spectra for high-intensity precursors were acquired for a shorter time (90,000 counts, 12 Hz) than low-intensity precursor ions (10,000 counts, 6 Hz) thus allowing for a balancing of maximal scan time and MS/MS spectral quality. As shown in Figure S1, the eight pooled quality control samples clustered in Principal Component Analysis (PCA) scores plots, and together with retention time CV% < 0.1 min, peak *m/z* values 3 mDa, and relative standard deviations of peak areas < 20%, there was good system stability, mass accuracy and reproducibility of the chromatographic separation during the whole LC–MS/MS sequence. In addition, intensity CV% of the identified compounds in pooled quality control samples are low (average 6%). PCA hotelling (T^2^) revealed one DHEA + subject as an outlier (D4) and was removed from further analysis (Figure S1).

### Compound identification

Structure identification was achieved via the following in MetaboScape (version 2.0): elemental composition was predicted via isotopic pattern following the rules (i) mSigma of MS1: 20 with tolerance of 5 ppm and (ii) MS2: 50 with tolerance of 2 mDa of the differential metabolites was searched against Bruker HMDB (Human Metabolome Database) using a precursor match of ± 10 mDa, minimum score of 400 and minimum match score of 250. Progesterone, glycerophosphocholine, linoleic acid and valine were structurally confirmed using chemical standards.

### Multiplex immunoassay analysis

Forty five  cytokines were detected and measured using ProCartaplex (EBioscience, CA, USA) as previously reported [BDNF, EGF, Eotaxin (CCL11), FGF-2 (FGF basic), GM-CSF, CXCL1 (GROα), HGF, IFNγ, IFNα, IL-1RA, IL-1β, IL-1α, IL-2, IL-4, IL-5, IL-6, IL-7, CXCL8 (IL-8), IL-9, IL-10, IL-12 p70, IL-13, IL-15, IL-17A, IL-18, IL-21, IL-22, IL-23, IL-27, IL-31, CXCL10 (IP-10), LIF, CCL2 (MCP-1), CCL3 (MIP-1α), CCL4 (MIP-1β), βNGF, PDGF-BB, PLGF, CCL5 (RANTES), SCF, CXCL12 (SDF1α), TNFα, LTA (TNFβ), VEGF-A, VEGF-D] [42]. Briefly, 5 μL of FFs were diluted with 5 μL Universal Dilution Buffer, and mixed with 50 μL of antibody-conjugated, magnetic beads in a 96 well DropArray plate (Curiox Biosystems, Singapore) and rotated at 450 rpm for 120 min at 25 °C while protected from light. Beads were internally dyed with different concentrations of two spectrally distinct fluorophores and covalently conjugated to antibodies against the 45 cytokines, chemokines and growth factors. The plate was washed three times with wash buffer (PBS, 0.05% Tween-20) on the LT210 Washing Station (Curiox) before adding 10 μL of secondary antibody and rotating at 450 rpm for 30 min at 25 °C protected from light. Subsequently, the plate was washed three times with wash buffer, and 10 μL of streptavidin–phycoerythrin added and rotated at 450 rpm for 30 min at 25 °C protected from light. The plate was again washed thrice with wash buffer; 60 μL of reading buffer was then added and the samples read using the Bio-Plex Luminex 200 (BioRad). The beads were classified by the red classification laser (635 nm) into its distinct sets, while a green reporter laser (532 nm) excites the phycoerythrin, a fluorescent reporter tag bound to the detection antibody. Quantitation of the 45 cytokines was then determined by extrapolation to a six or seven-point standard curve using five-parameter logistic regression modelling. Calibrations and validations were performed prior to runs and on a monthly basis respectively.

### Statistical analysis

GraphPad Prism 6 (GraphPad Software Inc.) was used for performing all statistical analyses. Data were checked for normal distribution using Kolmogorov–Smirnov test. Unpaired or paired t-test was performed, as appropriate, to determine statistical significance between groups form normally distributed data. Mann–Whitney U test was used for non-normally distributed data. For comparing more than three groups, the data were analyzed using ANOVA test, followed by the *t*-test with Bonferroni adjustment. *P* < 0.05 was considered significant. Metabolomic data was further analyzed by Principal Component Analysis (PCA) and Partial Least Squares Regression (PLSR) modelling (Unscrambler X version 10.1) after the normalization of data by first centering the data to the median and scaling it by division with the standard deviation. Full cross-validation was applied in PLSR to increase model performance and for the calculation of β-coefficient regression values [43]. Metabolites with β-coefficient regression values ≥ 1 are considered to have contribute significantly to the PLSR model. In this study, metabolites fulfilling both PLSR β-coefficient regression values > 1.2 and Student t-test *p* < 0.05 was considered as differential.

## Results

### Follicular fluid metabolomic analysis of POR patients

Significant increases were observed for clinical hormonal markers DHEA-S and free testosterone in the DHEA + patients compared to the DHEA- controls with treatment (*p* < 0.001; Figure S2). Estradiol, free testosterone and DHEA-S were significantly higher 4 months after treatment in the DHEA + patients (*p* < 0.001; Table 3).

**Table 3:** Comparison of AFC, ovarian volume and hormonal test at baseline and after 4 months of treatment in the DHEA group

Parameters^a^	4-month	Baseline	Relative mean difference, RMD^b^	*p*-value
	**(*n* = 24)**	**(*n* = 24)**	**(95% CI)**	
Basal FSH (IU/L)	6.8 (1.2–24.2)	8.7 (4.8–27.7)	0.78 (0.61–1.00)	0.052
Estradiol (pmol/L)	141.8 (56.0–290.0)	94.3 (37.0–320.0)	1.50 (1.24–1.83)	< 0.001
AMH (ng/ml)	0.6 (0.2–2.0)	0.6 (0.2–2.8)	0.99 (0.71–1.36)	0.932
Free Testosterone (pmol/L)	4.6 (2.0–9.5)	2.1 (0.8–14.3)	2.25 (1.74–2.91)	< 0.001
DHEA-S (umol/L)	14.3 (4.5–31.2)	4.3 (0.8–17.3)	3.35 (2.42–4.63)	< 0.001
Antral follicle count	4.4 (1–11)	4.6 (1–15)	0.96 (0.65–1.44)	0.854
Ovarian volume RO (cm^3^)	5.5 (0.9–14.3)	7.0 (1.9–24.0)	0.74 (0.47–1.16)	0.176
Ovarian volume LO (cm^3^)	5.2 (1.6–16.8)	4.8 (1.8–10.6)	1.10 (0.84–1.43)	0.472

^a^ Summarized by geometric mean (range)

^b^ Ratio of geometric means

From a total 2717 time-aligned features, an average of 903 features was chosen for auto MS/MS mode. From these, a total of 100 metabolites were identified via chemical standard confirmed HMDB [44]. An average of 65 MS/MS confirmed metabolites was identified per patient, which was similar in terms of metabolite identified in either DHEA- or DHEA + subjects (*p* = 0.8; range:59–76 metabolites), providing a global metabolome view of the FF metabolome in POR patients. The FF metabolome spanned three orders of magnitude, and was composed of a range of chemically diverse metabolites including, glycerophospholipids and derivatives (glycerophosphocholine, phosphatidylcholines), fatty acids (heptadecanoic acid, linoleic acid, vaccenic acid, myristic acid), cholesterols (isocaproic acid, 7-ketocholesterol), glucocorticoids (11-deoxycortisol or cortexolone, cortisol, corticosterone), hormones (17-hydroxyprogesterone, deoxycorticosterone, 11α-hydroxyprogesterone, 16-dehydroprogesterone, androstenedione, epitestosterone, progesterone, pregnenolone). Other metabolites included bile acids (3b-hydroxy-5-cholenoic acid, 3-oxocholic acid, glycocholic acid), peptides and derivatives (3-indolepropionic acid), lactones (delta-hexanolactone/caprolactone), lactic acid, vitamin D3 and sphingosine (Table 4).

**Table 4:** List of identified follicular fluid metabolites

No	HMDB No	Accurate mass	Theoretical mass	Compound Name	Chemical formula	Pathway
1	HMDB01859	151.0626	151.0633	Acetaminophen	C8H9NO2	Acetaminophen metabolism
2	HMDB04987	261.1206	261.1325	Alpha-Aspartyl-lysine	C10H19N3O5	Amino acid metabolism
3	HMDB03423/HMDB00641	146.0687	146.0691	D-Glutamine/L-Glutamine	C5H10N2O3	Amino acid metabolism
4	HMDB00714	179.0576	179.0582	Hippuric acid	C9H9NO3	Amino acid metabolism
5	HMDB00161/HMDB01310/ HMDB00271	89.0465	89.0477	L-Alanine/D-Alanine/Sarcosine	C3H7NO2	Amino acid metabolism
6	HMDB00641/HMDB03423	146.0687	146.0691	L-Glutamine/ D-Glutamine	C5H10N2O3	Amino acid metabolism
7	HMDB00687/HMDB00557/HMDB00172/HMDB01645	131.0941	131.0946	L-Leucine/L-Alloisoleucine/ L-Isoleucine/L-Norleucine	C6H13NO2	Amino acid metabolism
8	HMDB00696	149.0504	149.0510	L-Methionine	C5H11NO2S	Amino acid metabolism
9	HMDB00162	115.0629	115.0633	L-Proline	C5H9NO2	Amino acid metabolism
10	HMDB00883/HMDB00043	117.0784	117.0790	L-Valine/Betaine	C5H11NO2	Amino acid metabolism
11	HMDB00064/HMDB00766	131.0688	131.0695	Creatine/ N-Acetyl-L-alanine	C4H9N3O2	Arginine, proline, glycine and serine metabolism
12	HMDB00043/HMDB02141	117.0786	117.0790	Betaine/ N-Methyl-a-aminoisobutyric acid	C5H11NO2	Betaine Metabolism
13	HMDB01847	194.0796	194.0804	Caffeine	C8H10N4O2	Caffeine metabolism
14	HMDB01860	180.0642	180.0647	Paraxanthine	C7H8N4O2	Caffeine metabolism
15	HMDB02825	180.0638	180.0647	Theobromine	C7H8N4O2	Caffeine metabolism
16	HMDB00062	161.1048	161.1052	L-Carnitine	C7H15NO3	Fatty acid metabolism
17	HMDB00267/HMDB00805	129.0419	129.0426	Pyroglutamic acid/ Pyrrolidonecarboxylic acid	C5H7NO3	Glutathione metabolism
18	HMDB00017	183.0528	183.0532	4-Pyridoxic acid	C8H9NO4	Vitamin B6 metabolism
19	HMDB00995	312.2087	312.2089	16-Dehydroprogesterone	C21H28O2	Lipid metabolism
20	HMDB00502	388.2485	406.2719	3-Oxocholic acid	C24H38O5	Lipid metabolism
21	HMDB00308	356.2708	374.2821	3b-Hydroxy-5-cholenoic acid	C5H4N4O3	Lipid metabolism
22	HMDB00501	400.3339	400.3341	7-Ketocholesterol	C27H44O2	Lipid metabolism
23	HMDB00503	372.2659	390.2770	7a-Hydroxy-3-oxo-5b-cholanoic acid	C24H38O4	Lipid metabolism
24	HMDB00784	188.1041	188.1049	Azelaic acid	C9H16O4	Lipid metabolism
25	HMDB00015/HMDB01547	346.2131	346.2144	Cortexolone/Corticosterone	C21H30O4	Lipid metabolism
26	HMDB01547	346.2142	346.2144	Corticosterone	C21H30O4	Lipid metabolism
27	HMDB00063	362.2093	362.2093	Cortisol	C21H30O5	Lipid metabolism
28	HMDB00631	449.3131	449.3141	Deoxycholic acid glycine conjugate	C26H43NO5	Lipid metabolism
29	HMDB00573/HMDB03231	282.2564	282.2559	Elaidic acid/Vaccenic acid	C18H34O2	Lipid metabolism
30	HMDB00628/HMDB00234	288.2078	288.2089	Epitestosterone/Testosterone	C19H28O2	Lipid metabolism
31	HMDB00086	257.1026	257.1028	Glycerophosphocholine	C8H20NO6P	Lipid metabolism
32	HMDB00138	465.3096	465.3090	Glycocholic acid	C26H43NO6	Lipid metabolism
33	HMDB02259	270.2557	270.2559	Heptadecanoic acid	C17H34O2	Lipid metabolism
34	HMDB00666	130.0989	130.0994	Heptanoic acid	C7H14O2	Lipid metabolism
35	HMDB00689	116.0833	116.0837	Isocaproic acid	C6H12O2	Lipid metabolism
36	HMDB00673	280.2397	280.2402	Linoleic acid	C18H32O2	Lipid metabolism
37	HMDB00806	228.2088	228.2089	Myristic acid	C14H28O2	Lipid metabolism
38	HMDB00593	785.5943	785.5935	PC(18:1/18:1)	C44H84NO8P	Lipid metabolism
39	HMDB00847	158.1300	158.1307	Pelargonic acid	C9H18O2	Lipid metabolism
40	HMDB00253	316.2395	316.2402	Pregnenolone	C21H32O2	Lipid metabolism
41	HMDB01830	314.2239	314.2246	Progesterone	C21H30O2	Lipid metabolism
42	HMDB00792	202.1194	202.1205	Sebacic acid	C10H18O4	Lipid metabolism
43	HMDB00933	228.1335	228.1362	Traumatic acid	C12H20O4	Lipid metabolism
44	HMDB03231	282.2551	282.2559	Vaccenic acid	C18H34O2	Lipid metabolism
45	HMDB01877	144.1146	144.1150	Valproic acid	C8H16O2	Lipid metabolism
46	HMDB00876	384.3387	384.3392	Vitamin D3	C27H44O	Lipid metabolism
47	HMDB00182/HMDB03405	146.1048	146.1055	L-lysine/D-lysine	C6H14N2O2	Lysinuric protein intolerance
48	HMDB01923	230.0937	230.0943	Naproxen	C14H14O3	Naproxen action pathway
49	HMDB06344	264.1103	264.1110	Alpha-N-phenylacetyl-L-glutamine	C13H16N2O4	Phenylacetate Metabolism
50	HMDB00159	165.0784	165.1891	L-Phenylalanine	C9H11NO2	Phenylalanine metabolism
51	HMDB00097	103.0994	104.1075	Choline	C5H14NO	Phosphatidylcholine biosynthesis
52	HMDB00157	136.0380	136.0385	Hypoxanthine	C5H4N4O	Purine metabolism
53	HMDB00289	168.0278	168.0283	Uric acid	C5H4N4O3	Purine metabolism
54	HMDB00926	79.0421	79.0422	Pyridine	C5H5N	Pyridine biosynthesis
55	HMDB00975/HMDB00055	324.1052	342.1162	Trehalose/Cellobiose	C12H22O11	Pyrimidine metabolism
56	HMDB00300	112.0266	112.0273	Uracil	C4H4N2O2	Pyrimidine metabolism
57	HMDB00190/HMDB01311	90.0311	90.0317	L-Lactic acid/D-Lactic acid	C3H6O3	Pyruvate metabolism
58	HMDB00252	299.2818	299.2824	Sphingosine	C18H37NO2	Sphingolipid Metabolism
59	HMDB00374/HMDB00016/HMDB00920	330.2178	330.2195	17-Hydroxyprogesterone/ Deoxycorticosterone/11a-Hydroxyprogesterone	C21H30O3	Steroid biosynthesis
60	HMDB00929	204.0894	204.0899	L-Tryptophan	C11H12N2O2	Tryptophan metabolism
61	HMDB00197	175.0627	175.0633	Indoleacetic acid	C10H9NO2	Tryptophan metabolism
62	HMDB00183	208.0843	208.0848	L-Kynurenine	C10H12N2O3	Tryptophan metabolism
63	HMDB00158/HMDB06050	181.0735	181.0739	L-Tyrosine/o-Tyrosine	C9H11NO3	Tyrosine metabolism
64	HMDB02302	189.0782	189.0790	3-Indolepropionic acid	HMDB02302	Tryptophan deamination
65	HMDB01924	266.1620	266.1630	Atenolol	C14H22N2O3	Beta1-receptor inhibition
66	HMDB06115	106.0416	106.0419	Benzaldehyde	C7H6O	Oxidoreductase activity
67	HMDB00562	113.0586	113.0589	Creatinine	C4H7N3O	Arginine, proline, glycine and serine Metabolism/creatine catabolism
68	HMDB00453	114.0670	114.0681	Delta-hexanolactone	C6H10O2	Hydroxy acid lactonization
69	HMDB04983	94.0084	94.0089	Dimethyl sulfone	C2H6O2S	Methanethiol metabolism
70	HMDB01888	73.0524	73.0528	N,N-Dimethylformamide	C3H7NO	Tertiary carboxylic acid metabolism
71	HMDB00070	129.0785	129.0790	Pipecolic acid	C6H11NO2	Amino acid metabolism

### Altered POR follicular fluid metabolome in response to DHEA

**Fig. 1:**
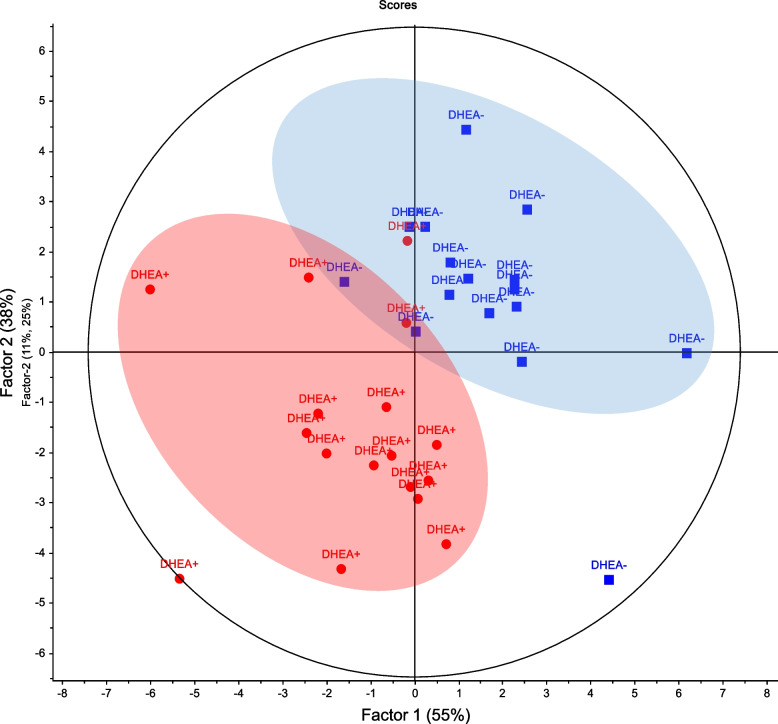
Partial Least Squares Scores plot of DHEA- and DHEA+ follicular fluid metabolome. Metabolomic data was median centred and scaled by division with the standard deviation. The follicular fluid metabolome distinguished POR subjects on DHEA supplementation (DHEA+, red) and without DHEA supplementation (DHEA-, blue)

**Fig. 2:**
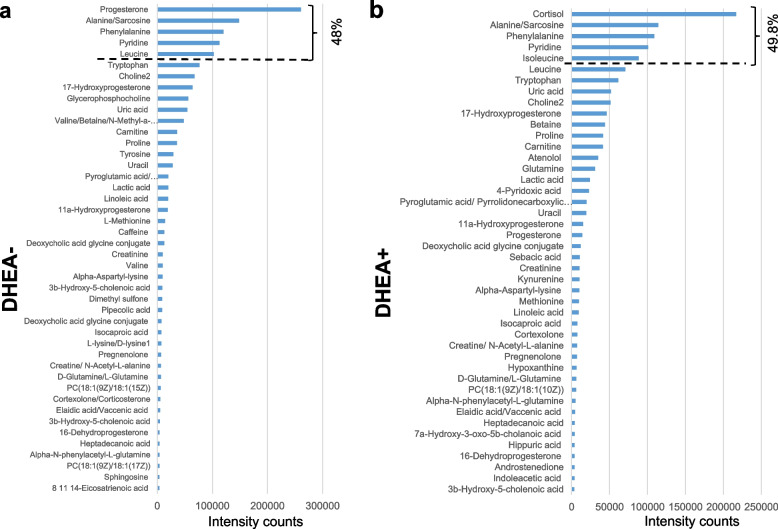
Histogram of follicular fluid metabolites in poor ovarian responders with and without DHEA supplementation. Follicular metabolome coverage and metabolite abundance as quantified by untargeted LC–MS/MS metabolomics in (**A**) DHEA- controls and (**B**) DHEA + poor ovarian response subjects. Metabolites were ranked according to their intensity counts

**Fig. 3:**
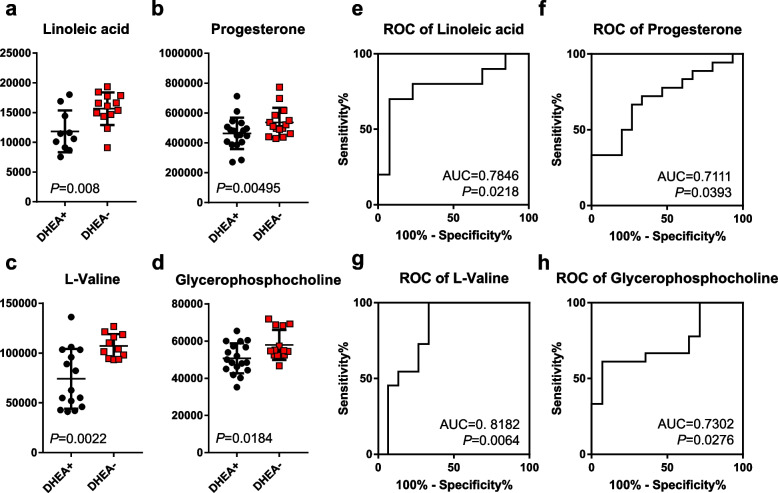
Significantly changed follicular fluid metabolites in DHEA+ and DHEA- patients. **a**-**d** Dot plots of significantly changed metabolites in poor ovarian responder patients. **e**-**f** Corresponding receiver operating curve (ROC) analyses of the metabolites. Area under curve (AUC) of the metabolites and their *P*-values are reported

Next, partial least squares-discriminant regression analysis (PLSR) was performed based on the overall features to compare the FF metabolomic profiles between the DHEA + and DHEA- groups. At metabolome-scale, the PLSR score plot showed that the FF metabolome clearly distinguished DHEA + patients from DHEA- patients (Fig. 1). Further analysis revealed that in DHEA- controls, progesterone was the most abundant FF metabolite, followed by L-alanine, L-phenylalanine, pyridine, L-leucine. The top five metabolites in terms of abundance collectively made up close to half (48%) of the DHEA- FF metabolome (Fig. 2A). In DHEA + , the FF metabolome profile of highly abundant metabolites was different, with cortisol as the most abundance metabolite, followed by L-alanine, L-phenylalanine, pyridine, L-isoleucine and L-leucine. These top six metabolites collectively made up ~ 49.5% of the DHEA + FF metabolome (Fig. 2B). Interestingly, pyridine, considered a non-endogenous metabolite (HMDB0000926) was found in such high abundance suggesting it came from the synthesis of DHEA [45]. The observed MS/MS spectra of pyridine at various eV matched very well with HMDB database (Figure S3), which suggested its correct identification. As a precursor to testosterone and estrogen, DHEA could be converted to testosterone, and aromatized to estrogen; in the case of POR, exogenous DHEA was proposed to increase androgens in promoting folliculogenesis and potentiate the effects of gonadotropins [8, 46, 47]. FF testosterone was detected in our metabolomics profiling, although the differences between DHEA + and DHEA- group were small [DHEA-: mean signal intensity = 2294.5; DHEA + : mean signal intensity = 2267.75 (testosterone), *p* > 0.05; Figure S3B]. Next, we screened for prominent metabolites that are differential with DHEA treatment in POR using the criteria of β-coefficient regression values > 1.2 and *p* < 0.05. Among the FF metabolites, glycerophosphocholine, linoleic acid, progesterone, and L-valine fulfilled the screening criteria and were significantly lower in DHEA + relative to DHEA- (Student’s t-test, *p* < 0.05–0.005; Fig. 3A-D). Although not significant, pregnenolone, a cholesterol metabolite and steroid that is upstream of DHEA metabolism, was detected only in DHEA + (6/18 subjects), and not DHEA- (0/16 subjects). Receiver operating characteristic (ROC) analyses of the four metabolites revealed area under the curve (AUC) ranging from 0.711 (progesterone), 0.730 (glycerophosphocholine), 0.785 (linoleic acid) and 0.818 (L-valine) (*p* < 0.05–0.01; Fig. 3E-H), suggesting the plausible utility of these FF metabolites in monitoring DHEA treatment. Additionally, linoleic acid and L-valine remained significantly lower in DHEA + (*p* < 0.05, *p* < 0.001 for both) when women with endometriosis (*N* = 5) were removed from analysis, strongly suggesting the significant effect of DHEA on these metabolites (Figure S4).

### Correlation of FF metabolites to biochemical hormones

In DHEA + patients, progesterone positively correlated with IGF-1 (Pearson r: 0.6757, *p* < 0.01); glycerophosphocholine negatively correlated with AMH (Pearson r: -0.5815; *p* < 0.05); linoleic acid correlated with estradiol and IGF-1 (Pearson r: 0.7016 and 0.8203, respectively; *p* < 0.01 for both; Figure S5A-D). Valine did not correlate with any biochemical hormones.

In DHEA- patients, few metabolites correlated weakly with estradiol, AMH,DHEA sulphate, serum-free testosterone, and IGF-1, with the exception of valine with serum-free testosterone (Pearson r: -0.8774; *p* < 0.0001; Figure S5E).

### FF cytokine profile in response to DHEA

**Fig. 4:**
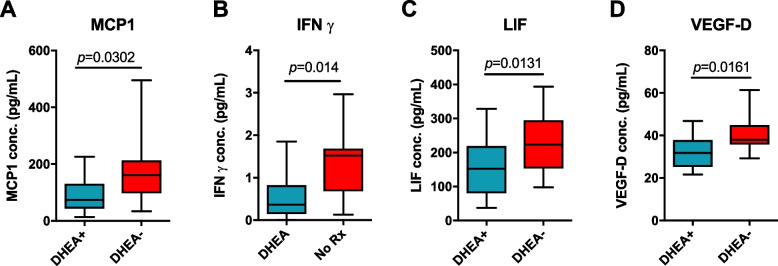
Significantly changed follicular fluid cytokines in DHEA+ and DHEA- patients. Among 45 cytokines, chemokines and growth factors measured by multiplex immunoassay, **A** MCP-1, **B** IFNg, **C** LIF and (**D**) VEGF-D were significantly lower in POR subjects with DHEA supplementation. Student’s t-tests were performed and *p*<0.05 is considered statistically significant.

Of the 45 cytokines, chemokines and growth factors investigated, 22 were detected in human FF, comprising of 10 cytokines (IFNγ, IL12p70, IL13, IL1b, TNFα, IL1Ra, IL5, IL7, IL10, IL18), 6 chemokines (eotaxin, IP-10, MCP1, MIP1β, SCF, SDF-1α) and 8 growth factors (bNGF, BDNF, EGF, HGF, LIF, PIGF, VEGF-A, VEGF-D). Among them, FF MCP1, IFNγ, LIF and VEGF-D were significant lower in DHEA + compared to DHEA- (*p* = 0.03, 0.014, 0.031, 0.0161 respectively; Fig. 4). No correlation was found between the significant cytokines and metabolites.

## Discussion

In this study, we report the first, and largest metabolome and large-scale cytokine study to-date of the FF of POR/DOR patients with DHEA supplementation. A total of 118 metabolites that included lipids, fatty acids, glucocorticoids, hormones, bile acids, peptides, and 22 cytokines were identified in the FF of POR/DOR patients. Among these, four FF metabolites, namely glycerophosphocholine, linoleic acid, progesterone, and L-valine varied significantly between DHEA + and DHEA- control subjects. This LC–MS/MS metabolomics study extends the human FF metabolome space in terms of characterization of its constituents, providing new insights into the complexities of oocyte development especially in POR women [48], as well as with DHEA supplementation [17–19, 49, 50].

The identification of four differential metabolites, glycerophosphocholine, linoleic acid, progesterone, and valine in this study may alert us to the metabolic effects of exogenous DHEA supplementation and plausibly using them to achieve improved outcomes [36, 51, 52]. Choline and derivatives are an emerging class of metabolites critical in developmental competence of fertilized oocytes [19], and glycerophosphorylcholine was found increased in the DHEA + group. Glycerophosphorylcholine is formed from the breakdown of phosphatidylcholine, and is an organic osmolyte, plausibly affecting concentrations of other constitutes of FF [16], and regulation of the diffusion of compounds into FF necessary for folliculogenesis and oogenesis [53]. PORs are known to exhibit a low diffusion of exogenous gonadotropin into FF, which is correlated with poor IVF outcomes [54]. It is conceivable that DHEA induced the metabolism of phosphatidylcholine to glycerophosphorylcholine. Progesterone is one of the key hormones for the progress of the first meiotic division in oocyte maturation, but changes to progesterone levels with DHEA supplementation has been controversial [55]. Our metabolomics study revealed for the first time that DHEA supplementation led to a decrease in FF progesterone levels, but to what impact lower progesterone induced by DHEA supplementation has on PORs remains to be investigated. Valine degradation has been previously reported in a proteomics study comparing competent versus incompetent buffalo oocyte proteome [56]. In a metabolomics study, valine metabolism was also identified in bovine cumulus and cumulus-oocyte-complex-conditioned media that undergo oocyte maturation [57]; although in both studies, valine was not directly detected in the omics profiling. In humans, degenerate oocytes or germinal vesicles that failed meiotically to reach metaphase II deplete valine more than competent oocytes. In other words, lower valine levels in culture media which is consistent with our results, and suggest plausible biological roles of valine in oocyte maturation. Interestingly, we noted a segregation of DHEA + patients with low and high level of valine, with the high valine group approaching concentrations of the DHEA- control group. Together with valine’s high AUC value in DHEA + , and that valine negatively correlated with testosterone, it is tempting to speculate that valine can be used as biomarker for monitoring individual DHEA supplementation. Linoleic acid is the most abundant polyunsaturated fatty acid in bovine [58] and human FF (Fig. 2), and varying concentrations of linoleic acid have reportedly different effects on oocyte maturation. At a concentration of 100 µM, linoleic acid added to maturation media inhibits bovine oocyte maturation and subsequent blastocyst development through increasing prostaglandin E_2_ concentration in the medium, decreasing intracellular cAMP, decreasing phosphorylation of the MAPK1 and AKT and inhibited germinal vesicle breakdown [58, 59]. Conversely, at concentrations at 50 µM or below, linoleic acid improved oocyte quality by increasing the content of neutral lipids stored in lipid droplets [59]. FF linoleic acid’s high AUC value and its correlation with estradiol suggest that can be another biomarker for titrating and monitoring individual DHEA supplementation.

The elevated DHEA-sulphate levels coupled with a lack of difference in FF testosterone with DHEA supplementation suggest the following possibilities in POR patients: (i) inadequate DHEA conversion to testosterone due to polymorphism in *SULT2A1, CYP19A1 and FMR1* genes [60], or (ii) long CAG repeats in androgen receptor gene which is linked to its lower transcriptional activity at the promoters of genes involved in the metabolism of DHEA to testosterone [61]. The former is unlikely: in a case–control study involving 94 subjects, androgen secretion was not impaired in pre-ovulatory follicles of POR compared to normal responders, and similar levels of follicular testosterone levels was reported [62]. However, ethnicity and genetic predispositions might play a role as Chinese women are reported to have higher free androgens and African American women lower [63], which might explain their differences in pregnancy rates in association with IVF than those observed among other ethnic groups. Conversely, long CAG repeats is associated with risk of POR and oocyte insensitivity to androgenic stimulation [64], thus hinting a tenable rationale on the observed similar FF androgen levels between the DHEA- controls and DHEA + subjects in this study and others [36]. The abundance of cortisol in DHEA + subjects is interesting, in particular that DHEA reduces circulating cortisol [65], indicating follicular versus systemic difference in how DHEA affects cortisol levels. In vitro, it was noted that DHEA suppresses cortisol activity [66], including the antagonist effects of DHEA on the anti-inflammatory responses induced by cortisol via glucocorticoid receptor-mediated pathways [67]. It is noteworthy that high FF cortisol levels found in fertilized IVF individuals compared to unfertilized individuals led to the postulation that oocyte exposure to cortisol is required with oocyte maturation [68]. The higher levels of FF cortisol observed in DHEA + subjects therefore argues for a compensatory response to modulate the ratio of the two hormones in an attempt to maintain a favourable FF response to mature oocytes [67].

In mouse models of polycystic ovary syndrome, treatment with DHEA resulted in increased production of cytokines such as serum TNFα, IL-6, IL12p70, and IFNγ [69, 70]. In this study, DHEA supplementation led to the reduction of FF IFNγ, LIF, MCP-1, and VEGF-D levels. It appears that DHEA modulates chemokines and growth factors in POR FF without a clear Th1 or Th2 immune response as proposed [52]. LIF or leukemia inhibitory factor is expressed in the ovary and controls follicular growth [71]. It was reported that LIF suppressed the growth of primary, secondary, and early antral follicles in cultured ovarian tissues [72]. The authors postulated that LIF produced in the late antral or graafian follicles is secreted to suppress the growth of the neighbouring primary, secondary, and early antral follicles as part of follicular growth [16]. Interestingly, when hCG is administered in rhesus macaques, at 12 h follicular LIF levels increase and induce follicle rupture and ovulation and decrease at 24 h [73]. In our study, the number of MII oocytes and oocytes trended higher in the DHEA + group, suggesting that the biological roles of LIF might have been achieved (follicular maturation and rupture) but inadequate to generate a clinically significant outcome. In vitro results suggested that follicles produce VEGF-A, with VEGF-A inducing the expanding vasculature to support the increased needs growing follicles [74]. The decrease in VEGF-A in DHEA + individuals is intriguing. Fisher et al*.*, described that in cultured follicles, the rise in VEGF-A levels in faster-growing follicles are dependent on FSH dose and oxygen tension [75]. There have been reports that DHEA inhibits oxygen consumption in neurons [76], tempting the postulation that DHEA inhibited oxygen consumption in follicle that subsequently led to lower production of VEGF-A in DHEA + individuals. Further, the lack of correlation between the significant cytokines and metabolites suggests that DHEA converting to steroids which subsequently modulate cytokine production within the follicular microenvironment is more complex than originally thought.

We note various strengths of this study. Firstly, due to the highly confident identification based on MS/MS, and mass accuracy of LC–MS/MS-based metabolomics, we were able to distinguish progesterone from DHEA, an advantage over interference-prone immunoassays that face a cross-reactivity bioanalytical problem [55]. Similarly, LC–MS/MS-based determination of androgens was preferred over immunoassays due to strong interference from DHEA [77]. We did not detect E1 and E2; because for phenolic hydroxyl group of estrogens to act as proton donors, the signal would be more sensitive in the negative ion mode electrospray ionization [78] than in the positive ion mode which was used in this study. Aside from previously reported constituents of FF such as linoleic acid [17, 22], amino acids [18], and steroids including progesterone, testosterone [79], this study also captured metabolic products of ovarian steroidogenesis, cholesterols and glucocorticoids in the FF. Secondly, this study is the largest-to-date, providing a global view, specifically of the effect of DHEA on the FF metabolome and cytokine profile in POR patients. Thirdly, the recruitment of women of relatively advanced age, low mean number of recovered oocytes (4.9) and low clinical pregnancy rate (5/52 or 9.6%), which contrast with other studies [22, 33] suggest the appropriate inclusion of POR patients.

Many studies of POR, including this study, suffer from important limitations. Firstly, POR patients represent a heterogeneous group of patients of different prognosis and with a range of patient and biochemical characteristics. To-date, no definition has been able to correlate their presentation with pregnancy or live-birth prognosis [80, 81], contributing to the difficulties in designing studies and trials to evaluate therapeutic modalities. Secondly, with respect to the similar clinical pregnancy rate between DHEA + and DHEA- subjects, this might be construed as a limitation in terms of sample size. However, various studies, including randomized clinical trials have not led to a clear outcome in terms of an improvement in clinical pregnancy rate, the number of oocytes retrieved, and embryos formed [31–37].

In conclusion, our study provided new insights to POR FF at the metabolome level, and as indicated from the FF metabolome analysis, exogenous DHEA to these patients altered the overall metabolome coverage and abundance to four metabolites. LC–MS global (untargeted) metabolomics analysis has provided the ability to reveal biologically relevant changes within a system, even at sensitive ranges before the precedence of gross morphological or phenotypical changes [82]. Hypotheses generated from this study included plausible mechanisms underlying DHEA metabolism, and the potential utility of glycerophosphocholine, linoleic acid, progesterone, and L-valine as markers to assess DHEA supplementation. Therefore, future directions include targeted quantitative LC–MS/MS approaches to be developed to detect and quantify four “responder” metabolites in approaches similar to those previously conducted on human peritoneal fluids and sera [83–85] to design treatment based on metabolomics profiles. Steroid hormones including testosterone should also be quantified via LC–MS/MS to establish baseline levels before commencing DHEA supplementation. Further, comparing POR and normal responders will provide further insights to the alteration of the FF metabolome, and reach a deeper understanding of underpinning pathophysiology to PORDisclosure of conflict-of-interest statement.

## Supplementary Information

**Additional file 1: Supplementary Figure 1.** Principle component analysis reveals DHEA+4 (arrow) as a potential outlier and was removed from subsequent analysis. **Supplementary Figure 2.** (a) MS/MS spectra of pyridine at increasing eV. (b) Follicular fluid testerosterone levels as measured by metabolomics. DHEA+, POR subjects on DHEA supplementation and DHEA- control without DHEA supplementation. **Supplementary Figure 3.** (a) Dot Plots of Linoleic acid and L-Valine after removal of women with endometriosis (*N*=5), (b) ROC curves of Linoleic acid and L-Valine after removal of women with endometriosis (*N*=5). **Supplementary Figure 4.** Histograms of estradiol, anti-müllerian hormone (AMH), DHEA-sulphate and insulin Growth Factor-1 (IGFBP-1) concentrations as determined by immunoassay. NS, not significant. **Supplementary Figure 5.** Scatter plots of (a) progesterone with IGF-1 (Pearson r: 0.6757, *p*<0.01), (b) linoleic acid with estradiol (Pearson r: 0.7016, *p*<0.01), (c) linoleic acid with IGF-1 (Pearson r: 0.8203, *p*<0.01), (d) glycerophosphocholine negatively correlated with AMH (Pearson r: -0.5815; *p*<0.05), (e) valine with serum-free testosterone (Pearson r: -0.8774; *p*<0.0001). Linear regression lines are shown.

## Data Availability

Available upon request.

## Abbreviations

DHEA: Dehydroepiandrosterone
ESI: Electrospray ionization
FF: Follicular fluid
FSH: Follicle stimulating hormone
hCG: Human chorionic gonadotropin
IVF: in vitro Fertilization
LC–MS/MS: Liquid chromatography-tandem mass spectrometry
POR: Poor ovarian responders
PLSR: Partial least squares-discriminant regression
PCA: Quality control (QC); Principal Component Analysis
