# Supplementary material for: Dehydroepiandrosterone supplementation and the impact of follicular fluid metabolome and cytokinome profiles in poor ovarian responders
Source: J Ovarian Res. 2023 Jun 2;16:107. doi: 10.1186/s13048-023-01166-6 (PMC10239139; doi:10.1186/s13048-023-01166-6)
Supplement: Supplementary file 1 — Additional file 1: Supplementary Figure 1. Principle component analysis reveals DHEA+4 (arrow) as a potential outlier and was removed from subsequent analysis. Supplementary Figure 2. (a) MS/MS spectra of pyridine at increasing eV. (b) Follicular fluid testerosterone levels as measured by metabolomics. DHEA+, POR subjects on DHEA supplementation and DHEA- control without DHEA supplementation. Supplementary Figure 3. (a) Dot Plots of Linoleic acid and L-Valine after removal of women with endometriosis (N=5), (b) ROC curves of Linoleic acid and L-Valine after removal of women with endometriosis (N=5). Supplementary Figure 4. Histograms of estradiol, anti-müllerian hormone (AMH), DHEA-sulphate and insulin Growth Factor-1 (IGFBP-1) concentrations as determined by immunoassay. NS, not significant. Supplementary Figure 5. Scatter plots of (a) progesterone with IGF-1 (Pearson r: 0.6757, p<0.01), (b) linoleic acid with estradiol (Pearson r: 0.7016, p<0.01), (c) linoleic acid with IGF-1 (Pearson r: 0.8203, p<0.01), (d) glycerophosphocholine negatively correlated with AMH (Pearson r: -0.5815; p<0.05), (e) valine with serum-free testosterone (Pearson r: -0.8774; p<0.0001). Linear regression lines are shown. [file 13048_2023_1166_MOESM1_ESM.pptx]

## Slide 1
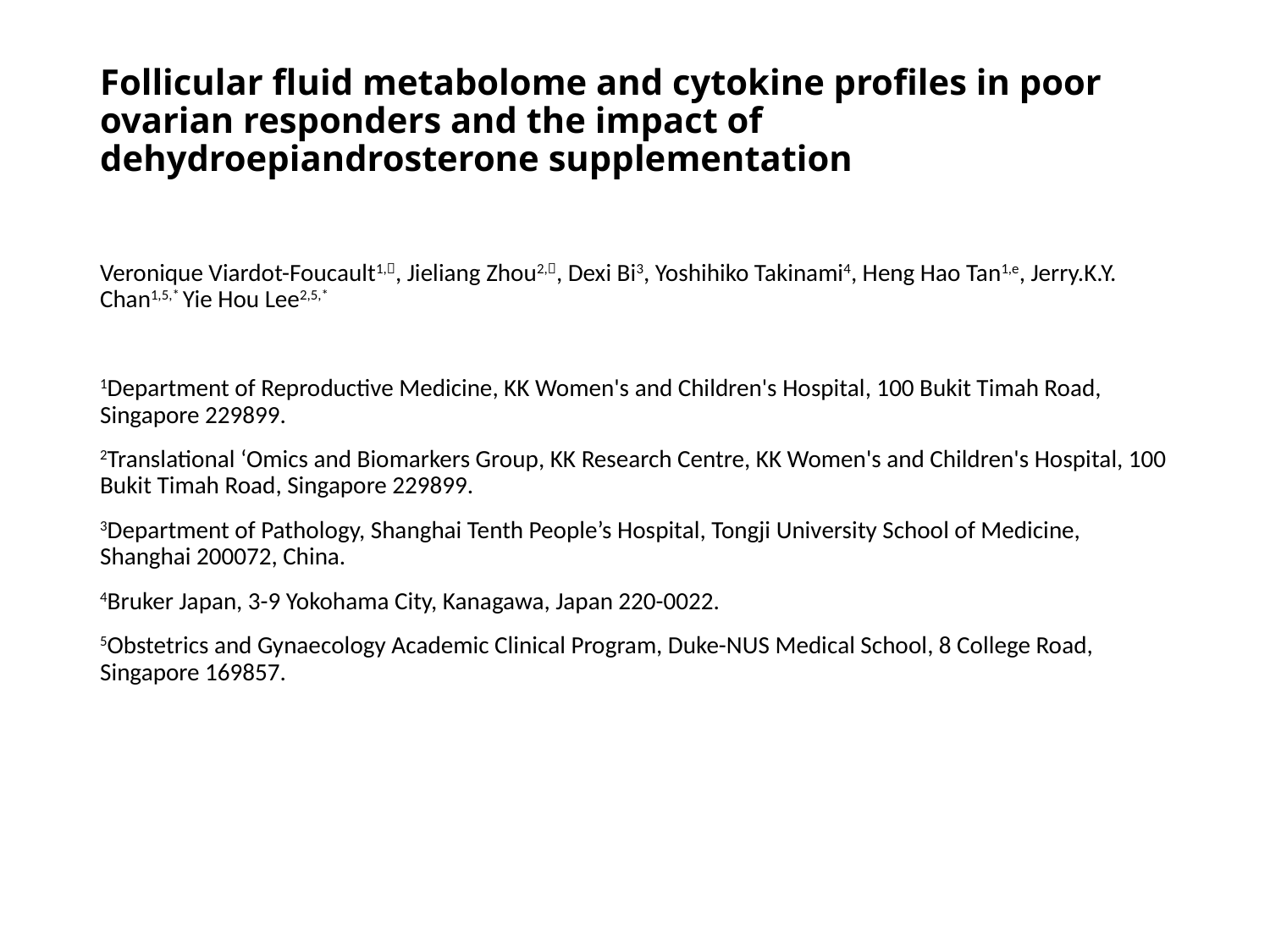

# Follicular fluid metabolome and cytokine profiles in poor ovarian responders and the impact of dehydroepiandrosterone supplementation
Veronique Viardot-Foucault1,, Jieliang Zhou2,, Dexi Bi3, Yoshihiko Takinami4, Heng Hao Tan1,e, Jerry.K.Y. Chan1,5,* Yie Hou Lee2,5,*
1Department of Reproductive Medicine, KK Women's and Children's Hospital, 100 Bukit Timah Road, Singapore 229899.
2Translational ‘Omics and Biomarkers Group, KK Research Centre, KK Women's and Children's Hospital, 100 Bukit Timah Road, Singapore 229899.
3Department of Pathology, Shanghai Tenth People’s Hospital, Tongji University School of Medicine, Shanghai 200072, China.
4Bruker Japan, 3-9 Yokohama City, Kanagawa, Japan 220-0022.
5Obstetrics and Gynaecology Academic Clinical Program, Duke-NUS Medical School, 8 College Road, Singapore 169857.

## Slide 2
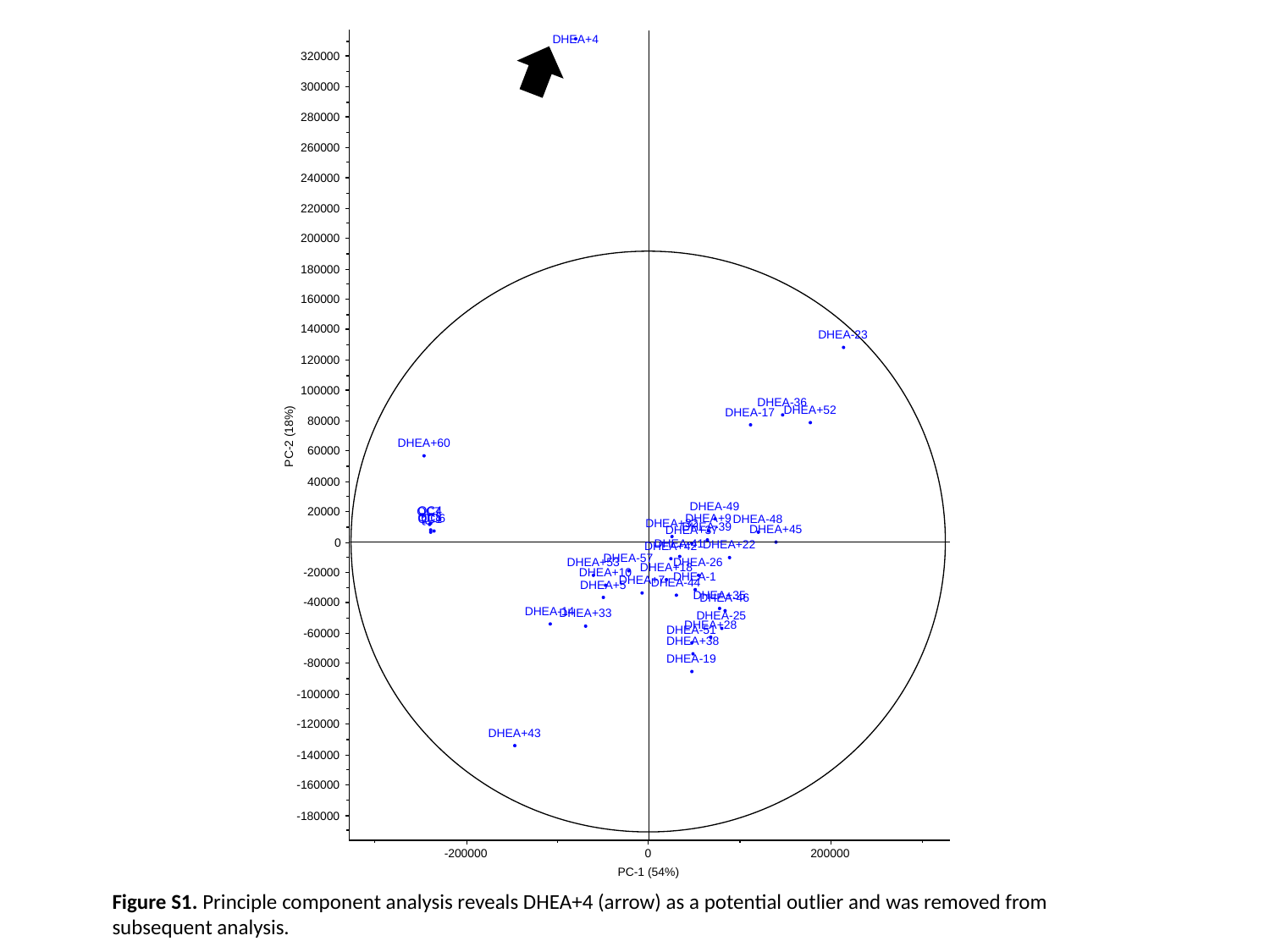

Figure S1. Principle component analysis reveals DHEA+4 (arrow) as a potential outlier and was removed from subsequent analysis.

## Slide 3
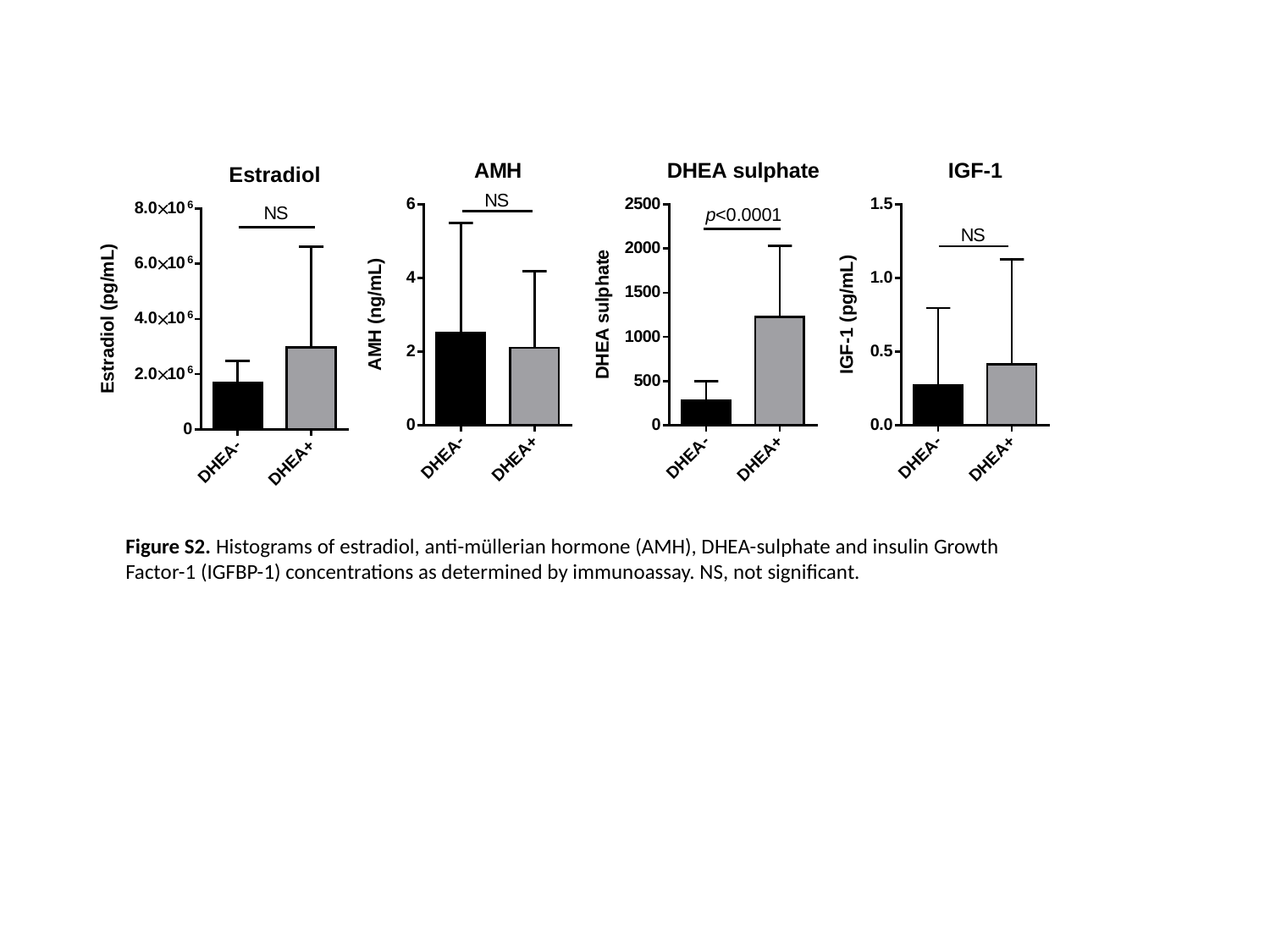

Figure S2. Histograms of estradiol, anti-müllerian hormone (AMH), DHEA-sulphate and insulin Growth Factor-1 (IGFBP-1) concentrations as determined by immunoassay. NS, not significant.

## Slide 4
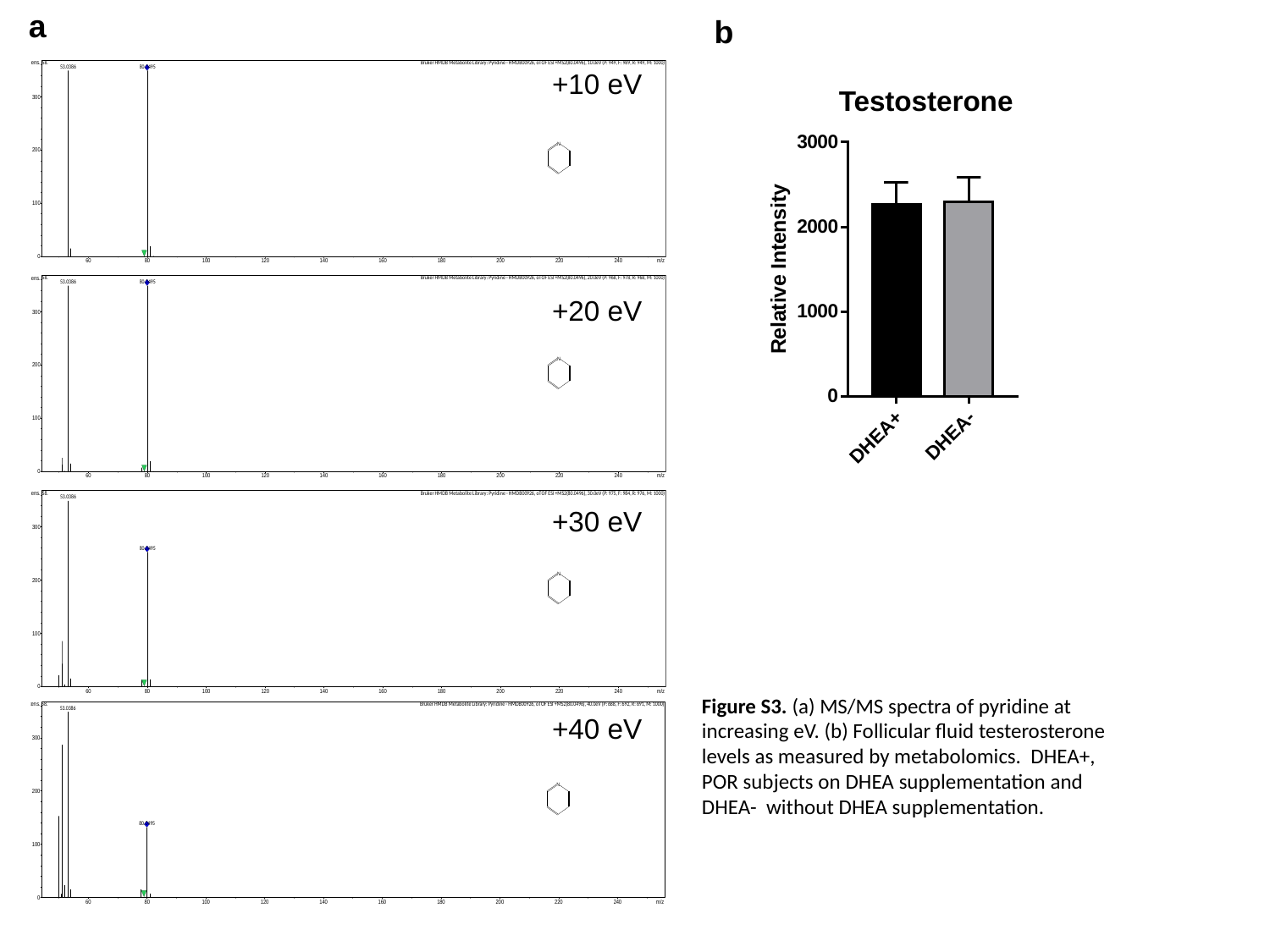

a
b
+10 eV
Testosterone
+20 eV
+30 eV
Figure S3. (a) MS/MS spectra of pyridine at increasing eV. (b) Follicular fluid testerosterone levels as measured by metabolomics. DHEA+, POR subjects on DHEA supplementation and DHEA- without DHEA supplementation.
+40 eV

## Slide 5
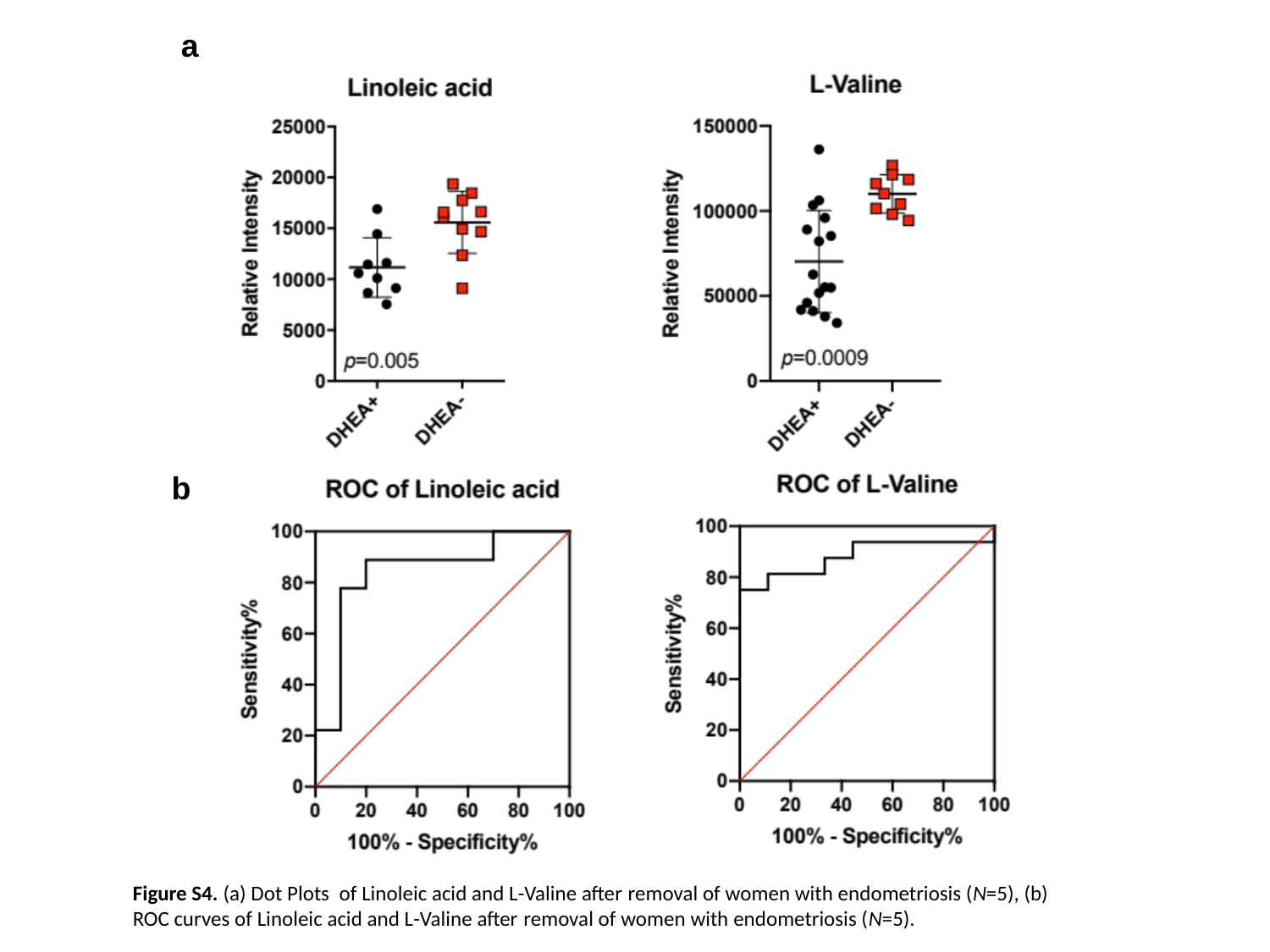

a
b
Figure S4. (a) Dot Plots of Linoleic acid and L-Valine after removal of women with endometriosis (N=5), (b) ROC curves of Linoleic acid and L-Valine after removal of women with endometriosis (N=5).

## Slide 6
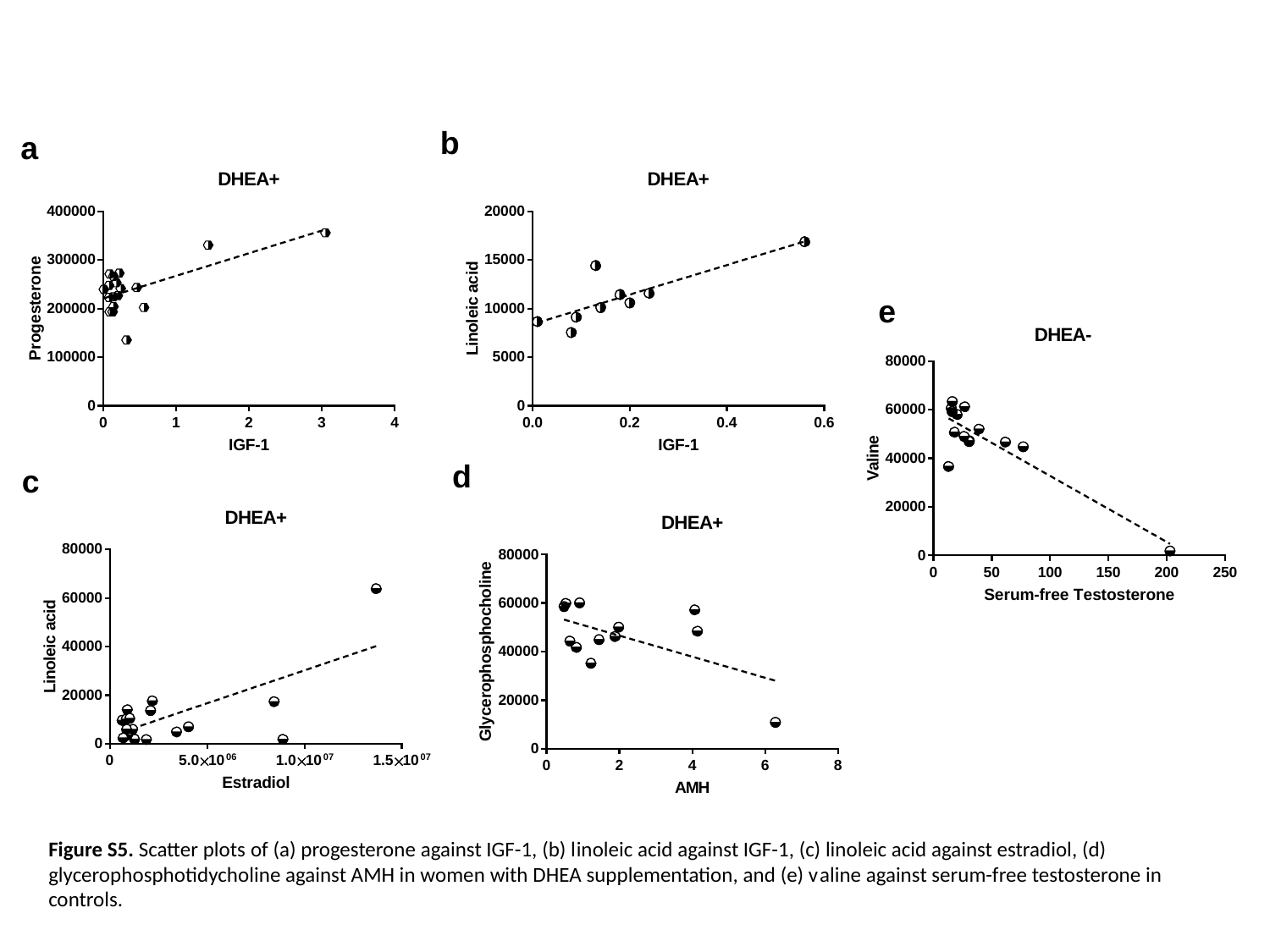

b
a
e
d
c
Figure S5. Scatter plots of (a) progesterone against IGF-1, (b) linoleic acid against IGF-1, (c) linoleic acid against estradiol, (d) glycerophosphotidycholine against AMH in women with DHEA supplementation, and (e) valine against serum-free testosterone in controls.
